# The influence of local government competition on residents’ perceptions of social fairness—Evidence from China

**DOI:** 10.3389/fpsyg.2022.1066691

**Published:** 2022-11-25

**Authors:** Junling Yi, Jingling Li

**Affiliations:** ^1^Department of Politics and Public Administration, Wuhan University, Wuhan, China; ^2^Department of Accounting, School of Accounting, Hubei University of Economics, Wuhan, China

**Keywords:** residents’ perceptions of social fairness, local government competition, income disparity, basic public goods supply, corruption

## Abstract

Social fairness has been one of the important issues and pursuits in the course of human history since ancient times, and the promotion of social fairness has become a social consensus. Based on the data from the years 2013, 2015, and 2017 Chinese General Social Survey (CGSS), an ordered probit model was constructed for empirical testing to explore the effect of local government competition on residents’ perceptions of social fairness and its internal mechanism. The research results show that: (1) Local government competition expands residents’ perceptions of social unfairness. (2) Local government competition increases residents’ perceptions of social unfairness through the paths of increasing residents’ income disparity, crowding out the supply of basic government public goods, and increasing corruption. (3) Local government competition has a significant negative effect on the perceptions of social fairness of the middle-income as well as the high-income but does not affect the low-income. The inhibitory effect of local government competition on the perceptions of social fairness of residents in urban as well as eastern regions is more significant than that in rural and central and western regions. This study has important practical implications for promoting common prosperity to build a harmonious, fair, and democratic modern welfare state and improving the governance capacity of local governments.

## Introduction

The report of the 19th National Congress of the Communist Party of China pointed out: “fairness and justice are the inherent requirements of socialism and an important governing goal.” The central government has paid more and more attention and introduced various social policies to the construction of a socialist country with fairness. However, there are still a series of social inequities while the economy is booming. Since the reform and opening up, China’s economy has made great progress. Economic development is not only closely related to the reform policy of modernization but also inseparable from the government governance system. As a “government-led” country, China has obvious characteristics of government intervention in the economy. Local government competition has contributed to the rapid development of China’s economy (Zhou, 2003). The competition of local governments has brought positive effects such as economic growth and efficiency improvement. At the same time, it has also expanded the social unfairness of residents under the influence of the experience of “the negative effects of high welfare” and “the high efficiency of the market system.” From the perspective of the top-level political system, Under the pressure of political performance appraisal in the economic championship, local governments adopt informal behaviors to compete to achieve the goal of GDP growth, which leads to many social problems affecting social fairness, such as the gap between the rich and the poor, the lack of government in the field of social welfare, and the corruption of public officials. The perceptions of social fairness are the subjective value experience of residents. The objective behavior of individual residents will be affected by their subjective consciousness. The stronger people’s perceptions of social fairness are, the higher the people’s perceptions of social trust will be, and the more positive pro-social behavior they will show (Van Prooijen et al., 2006).On the contrary, a low perception of social fairness will increase the group’s perceptions of social conflict (Li et al., 2012), leading to a decrease in intergroup trust and a lack of contact or even stimulating social conflicts affecting social stability (Alesina and Eliana, 2002). It is particularly important to clarify the extent of the impact of local government competition on residents’ perceptions of social justice and the underlying mechanisms.

Research on residents’ perceptions of social fairness has been relatively abundant, mainly focusing on the influencing factors and mechanisms. The current studies on the factors influencing the perceptions of social fairness are economic development (Feng and Su, 2021), income level distribution (Ma and Liu, 2010), anti-corruption (Li and Zhang, 2021), government basic public service supply (Li et al., 2018), and fiscal expenditure structure (Sun and Zhang, 2004), etc. But there are no studies on the influence of local government competition factors on residents’ perceptions of social fairness. To address this gap, this paper selects the observation perspective of a local government competition to study its specific effect on the perceptions of residents’ social fairness. In addition, in the discussion on the influence mechanism of residents’ perceptions of social fairness, sociologists have focused on the theory of structural position and the theory of relative deprivation. The objective social structural position theory holds that objective social inequality is positively related to a subjective perception of social justice, people in higher structural positions have a higher perception of social justice (Weng, 2010). However, this conclusion does not apply to all phenomena regarding the perceptions of social justice. For example, Moting (2009) research showed that some residents with higher class positions react more strongly to social inequality instead. The relative deprivation perceptions theory refutes the structural position theory of social equality. It is believed that the perceptions of social fairness mainly come from the residents’ perceptions of relative deprivation through “social comparison.” The lower the level of relative deprivation is, the stronger the perceptions of social equality are. Both of these theories can enable us to explore the mechanisms that influence residents’ perceptions of social fairness, and greatly deepen people’s understanding of social fairness. But they both start from the micro-level of the individual and ignore that the macro-level of the government is also an important subject and one of the influencing factors in achieving social fairness. The behavior of local government plays a significant role in maintaining social fairness, but this assumption is rarely explored.

Therefore, given the above analysis, this paper combines the years from 2013, 2015, and 2017 China General Social Survey (CGSS) micro-data and government-level macro-data set to conduct an empirical test using an ordered probit model to explore the effect of local government competition on residents’ perceptions of social fairness and its intrinsic mechanism of action. The results of the study showed that local government competition expands residents’ perceptions of social unfairness, mainly through three channels: widening the gap between rich and poor residents, crowding out basic public services such as education, health care, and social security, as well as increasing corruption. In addition, the heterogeneity test found that local government competition has an obvious negative effect on the perceptions of social fairness of middle-income and high-income groups, but has no impact on the perceptions of social fairness of low-income groups. Compared with rural residents and residents in the central and western regions, local government competition has a more significant inhibiting effect on the social justice of urban residents and residents in the eastern region. Once the gap between the rich and the poor and the resident’s perceptions of social fairness are formed, it is often difficult to reverse it. The participation of existing vested interests will increase the resistance to governmental reform when the fact or class is solidified, so income distribution should be carried out in the process of economic development. A good social fairness mechanism should be established so that people can share the fruits of economic development. This study has important implications for dealing with the imbalance between economic and social development, the excessive gap between the rich and the poor, and social unfairness, as well as for strengthening the governance capacity of local governments and promoting common prosperity to further build a fair and democratic modern state.

## Institutional background, theoretical analysis, and research hypothesis

### Institutional background

Local government competition refers to the process in which each local government, based on fiscal decentralization, adopts corresponding public policies to compete for resources to maximize the interests of each jurisdiction. The forms of participation include tax competition, public goods competition, and institutional competition, manifested as horizontal competition, vertical competition, and scalar competition (Huang and Zhou, 2001). The research on local government competition originated in the West. In terms of public goods competition effects, Tiebout (1956) and Oates (1972) et al. argue that free-moving residents will migrate to jurisdictions which might satisfy them. And local governments will adopt a series of efficient fiscal policies and public goods measures to meet residents’ demands to prevent the outflow of residents and capital, thus increasing the public goods supply efficiency of jurisdictional governments. In terms of the economic effects of local government competition, Van Prooijen et al. (2006) argue that local governments compete to improve the business environment through institutional innovation, tax incentives, and other proven administrative initiatives to attract the inflow of other jurisdictional resources to raise fiscal revenues and promote the economic development of their jurisdictions when resources are within limited availability. However, some studies take a negative attitude towards local government competition, Zodrw and Mieszkowski (1986) constructed a model to find that land is not mobile while capital is mobile across jurisdictions, jurisdictional governments will seek to maximize land rents and will compete fiercely for capital taxes. As the number of competing jurisdictions continues to increase capital taxes will race to the bottom eventually leading to too low a level of tax revenue in each jurisdiction, thus causing residents to doubt local government administrative systems and government public. This “destructive local competition” can lead competing local governments into a “prisoner’s dilemma” of avoiding business flights from residents and failing to provide sufficient public goods.

Although different from the federal state structure of the developed countries in the West, as long as there are multiple levels of government decentralization in any type of government, there will inevitably be competition between sub-governments for their interests. Competition among local governments also exists in China. During the transition of the domestic economy from a planned economy to a market economy, the central government uses fiscal transfer policies and government performance appraisal systems to control local governments, which in turn assume governmental responsibilities through certain fiscal expenditures and tax revenues, as well as the corresponding independent authority. Local governments play political games with the central government. Meanwhile, local governments develop local economies and stabilize their jurisdictions to obtain more administrative resources and a better institutional environment. At the same time, local governments strengthen their competitiveness through technological innovation and continuous improvement of infrastructure to compete with their counterparts in attracting resources and competing for talent and economic markets. In this process of progressive restructuring, China has developed a pattern of vertical competition among different levels of government and horizontal competition among peer governments. More characteristic of China, as a developing country, economic development has always been a priority. Thus a political environment has been created in which the promotion prospects of local government officials are linked to regional economic performance based on an assessment of the jurisdiction’s GDP. The political pressure for promotion internalizes the behavior of local government officials. Each local government official tries to integrate as much as possible the economic and political resources under his or her control and influence to promote the economic growth of the region (Zheng et al., 2005). Under China’s highly centralized administrative system of personnel power, officials at the provincial, municipal, county, and township levels of government are in a political promotion or political tournament in which they compete for economic performance and fiscal revenue within their jurisdictions (Zhou, 2004).

### Theoretical analysis and research hypothesis

#### Local government competition, income disparity, and residents’ perceptions of social fairness

Local government competition leads to the inequality of residents’ income by widening the income gap between urban and rural areas and regional income as well as the intervention in the market. The meaning of fairness is richer, the fairness of power, the fairness of the outcome, the fairness of opportunity, etc. But at this stage, the main discussion is the fairness in the field of distribution, mainly reflected in the income gap between different social classes (Li and Xu, 2021). Changes in income disparity in China are closely related to the historical process of urban–rural social changes and economic policy adjustments brought about by government-led economic growth and market-oriented reforms. The current income disparity among Chinese residents is mainly concentrated between regions and between urban and rural areas (Xie and Zhou, 2014). And the important factors affecting income inequality include the government and the market (Cai and Yue, 2016).

First, local government competition will widen the income gap between urban and rural residents. The “urban–rural dualistic economic structure” began to form as a result of the long-standing “catch-up” strategy and the urban bias of “planned allocation” of resources before the reform. Government officials have incentives for political promotion, and the “political tournament” around local GDP growth directly influences the behavior of local government officials. Under this pattern of local government competition, the government-dominated economic system directly influences changes in the urban–rural income gap through the allocation of public financial resources and the urban bias of economic policies. The government’s mechanism of targeting investments and choices to “high-value, high-income” enterprises and urban areas with good development conditions hinders the efficient allocation of capital, land, and other factors, as well as the “power allocation” that relies on policy protection and resource management. It distorts the way of income sharing and increases income inequality (Chu and Jin, 2013). At the same time, the “profit-seeking” nature of market micro-enterprises makes them reluctant to invest in agriculture and rural areas, resulting in long-term neglect of agricultural and rural development and a significant widening of the urban–rural income gap. Secondly, local government competition can aggravate the income gap between regions (Zhang, 2017). Not all governments in the local government competition system are competitive and rational at the beginning, but a small number of local governments are full of competitive spirit. They are the first to innovate through “institutional competition” to obtain more resources to improve the economy and the welfare of residents in their jurisdictions, which is the beginning of regional economic growth and disparities (Jin, 2017). As the competition continues, other provinces are forced to join the competition passively as the disparity between jurisdictions widens. When a province or municipality becomes a relatively independent body of economic or political interests, which is to their advantage. The local government that wins the competition establishes a cycle of power and economic circulation, using the administrative power and economic advantages it holds to erect barriers to circulation for localist protection as well as regional segmentation and blockade, which in turn increasingly increases economic inequality among regional residents.

The socioeconomic status hypothesis and objective social structure location theory consider economic income disparity as an important factor contributing to the decline in residents’ perceptions of social fairness. From an individualistic perspective, socioeconomic status affects an individual’s ability to access and control resources, as well as an individual’s attribution of fairness in his or her access to resources and distribution outcomes (Tian and An, 2019). The socioeconomic status brought about by the economic income gap also determines a certain degree of the high or low social structural position of the population, with those who have higher socioeconomic status and are in a higher structural position having a higher perception of social fairness (Cheng, 2007). Excessive income disparity leading to a highly unequal income structure can threaten social fairness and directly inhibit the public perceptions of social justice (Ling and Liu, 2018). Excessive income disparity brings a psychological feeling of unfairness to individual residents through subjective perceptions, which ultimately undermines social fairness.

Given the above analysis, this paper proposes research hypothesis H1: Local government competition amplifies the perception of residents’ social unfairness by widening the income gap among residents.

#### Local government competition, government supply of basic public goods, and residents’ perceptions of social fairness

The government uses taxpayers’ money to provide its public goods but has a monopoly on the supply of public goods. Local governments and administrative officials have to compete as long as residential and capital factors can flow through separate administrative jurisdictions in the country (Ke and Shi, 2000). The Chinese government is economically investment-oriented because of the exceptionally intense competitive dynamics of local governments under the Chinese political and economic system that uses GDP growth as a political promotion assessment mechanism. Local governments tend to distort the structure of government fiscal spending on infrastructure development and public services for the sake of GDP growth (Fu and Zhang, 2007). Education fairness and health care fairness are the starting point and prerequisite for social fairness and justice. The government invests too many financial resources in infrastructure and productive public goods and squeezes out non-productive public service expenditures such as education, medical care, and social security, which are closely related to people’s welfare (Zhang and Chen, 2006). The provision of basic public services can, on the one hand, provide more opportunities for the lower class to improve their human capital and, on the other hand, improve the risk-taking ability of all social classes to promote social mobility, thus calming people’s perceptions of social unfairness (Zhang and Fu, 2009). The financial shortage of public services *per capita* in China has led to low perceptions of social fairness.

Competition for local government officials in China is fierce (Lou, 2010). The promotion and re-election of local government officials in China do not come primarily from elections but from central government appointments. People in the jurisdiction lack channels to express their needs for public goods through the selection of local government officials. The pyramidal power structure has led to a large number of local government officials in China, which makes it difficult for officials to be promoted and the degree of competition is strong. In addition, to the short tenure of officials, to quickly achieve “performance” and “promotion” within the limited tenure, local government officials tend to pay attention to short-term utility and give up investing in public services (Wang, 2021). Investment in public services is difficult to see the return in the short term before the long-term gain. Thus, the competition among local officials will lead to the government’s emphasis on the economy rather than people’s livelihoods further aggravating the misalignment of government functions, directly causing the local government’s shortage of basic public services and public goods supply (Fu, 2010). In addition, based on the Western public choice school of thought, competition among local governments is expected to lead to more efficient public goods. The basic premise of these theories is that capital and residents will choose different jurisdictions using a “vote with their feet” approach, and the models generally set local government competition in a single institutional setting assuming that market capital or residents are perfectly mobile. However, due to its particular historical origins, China’s household registration system restricts the free movement of residents, preventing them from moving to provinces and cities with better public services. This administrative system, which prevents the free movement of people (Zhang and Zhao, 2017), is fundamentally at odds with the Western public choice school of thought. Some residents who work or live in cities with better public services may not be able to enjoy the welfare policies or suffer unfair treatment because they do not have a local hukou (household registration). The apparent resource asymmetry and differential treatment of power and opportunity in education, health care, and social security benefits can exacerbate residents’ perceptions of social unfairness.

Given the above analysis, this paper proposes research hypothesis H2: Local government competition amplifies the perception of social inequity by crowding out essential government public goods.

#### Local government competition, corruption, and residents’ perceptions of social fairness

Local government competition tends to increase the level of political corruption. Based on the principal-agent theory, local governments have a dual fiduciary-agent relationship with the central government and citizens. Due to information asymmetry and fiscal decentralization, local governments have a lot of financial control and discretionary power over local affairs. Local government officials have huge economic and administrative resources that make them face many temptations. Some private sectors or interest groups try to find ways to find rent and entice and bribe local government officials to use their resources and political power to “facilitate and open the back door” for themselves (Pan and Wu, 2019). The powerful position of local governments in China’s social structure has led to less oversight and checks and balances, increasing egoistic government officials’ adverse selection and opportunistic behavior. The ensuing selfish government officials engage in power and money deals to maximize political rents (Li, 2016). Especially when local governments invest in massive infrastructure construction or large capital investments for GDP competition. Because construction projects or capital investments involve large, complex, and non-standard activities whose quality is difficult to assess. They allow maximum rent-seeking and a large amount of corruption is hidden in the field of infrastructure investment (Yu et al., 2021). A more corrupt official or government will support the government’s financial choices in projects that favor rent-seeking such as infrastructure and capital projects. Empirical evidence suggests that bribery of officials with discretionary authority in government contracting and regulatory-related industries is common. Local government competition can lead to an increase in the size of government, which not only makes it more difficult to monitor local governments but also reduces the salary income of local government officials, making them more likely to engage in profit-seeking activities such as administrative “fishing” (Zhou and Tao, 2009). In addition, some officials in backward areas feel hopeless in the competition for promotion in local government, and they will adopt the attitude of “breaking the pot,” hoping to use their power to make up for the loss of money and power when they cannot be promoted, which will also lead to corruption (Tang et al., 2013).

Classical fairness theory assumes that people have all the information about their payoffs and rewards and that they can make a fair judgment through rational calculation and comparison (Adams, 1963). People do not have such information, so emotions play a role in judging fairness instead of information, and this emotional dynamics mechanism considers social fairness as a special judgment mechanism. Fairness is the result of individual judgment, and the judicial process is largely influenced by individual intuition and emotion. Thus, it can be seen that residents’ real social justice is not entirely based on rationality and calculation to judge whether society is fair or not (Lin, 2001), but often has obvious emotions, intuition, emotions, and other subjective emotional association factors, so we should not ignore such emotional factors in the analysis of the formation mechanism of residents’ perceptions of fairness. The impact of corruption on the perceptions of social justice corresponds to the cognitive and emotional dynamics of the formation of the perceptions of social justice. Corruption not only undermines the functioning of the administrative system (Anderson and Tverdova, 2003) but also shakes the socioeconomic foundation (Mauro, 1995). It is a persistent problem in the political system because of its complex and hidden means, and it is often difficult for the general public to know the specific amount, means, and input–output ratio of corruption. However, the massive exposure and in-depth disclosure of corruption information may allow the general public to directly learn about specific cases that are otherwise unknown to them, which gives the public a concrete outlet for their emotions but only a glimpse of the tip of the iceberg. According to the Information Uncertainty Management Model, in situations of information uncertainty, people often try to judge fairness by other means. This is where emotions play a significant role, and information uncertainty is one of the mediating moderators (Kees, 2003). Corruption exacerbates the influence of emotions through uncertain information contexts leading to more pronounced and intuitive perception of social unfairness. While the public can accept wealth or class disparities caused by differences in individual talents and abilities, it is often more difficult to tolerate corruption outside the system, especially when it is a power factor. Studies have shown that the “relationship economy” and official corruption are the main factors contributing to the perceptions of social unfairness (Li, 2006). Because “attribution preference” is more sensitive to people’s judgments about social justice, the stronger the perceptions of distributive justice are when people attribute the cause of social inequality to personal factors, and the stronger the perceptions of distributive injustice are when people attribute the cause outward to power and the system.

Given the above analysis, this paper proposes research hypothesis H3: Local government competition amplifies the perception of social inequity by fostering corruption. Given the above analysis.

In short, the central government cedes certain economic and fiscal administrative powers to local governments, establishes a performance appraisal system centered on economic growth, and makes local governments pay attention to economic development through personal incentives such as the selection and promotion of officials. However, in the relative performance assessment of the incentive structure of the Chinese government’s governance political tournament model, government officials may deviate from the interests of their constituents according to their interests, or excessive competition among local governments may weaken the market mechanism and lead to chaos and distortion of resource allocation, resulting in an excessive gap between the rich and the poor, lack of social public utilities, and government corruption. This reduces the residents’ sense of the social public.

Given the above analysis, this paper proposes research hypothesis H4: Local government competition will amplify residents’ sense of social inequity.

## Data and methods

### Data sources

The individual-level data used in this paper are from the years 2013, 2015, and 2017 China General Social Survey (CGSS). It is a large-scale comprehensive social survey designed and implemented by the National Survey Research Center at Renmin University of China which uses a multi-stage stratified probability proportional sampling and covers 31 provincial administrative units (autonomous regions and municipalities directly under the Central Government, excluding Hong Kong, Macao, and Taiwan) in mainland China, except for the Tibet Autonomous Region. The surveyed content involves many aspects of the respondents, such as background information, marital and family status, work and income, attitude, and behaviors, indicating a strong representativeness and credibility. Economic data at the regional level were obtained from the China Statistical Yearbook. The resident population data at the regional level were obtained from the Statistical Bulletin on National Economic and Social Development released by each province, autonomous region, and municipality directly under the Central Government. Corruption data at the regional level were collected manually from the content of each provincial procuratorate’s chief procurator’s reports to their corresponding provincial people’s congresses in the China Procuratorial Yearbook in previous years. For better analysis, this paper collated the initial data according to the following methods: (1) Samples with missing information were excluded. (2)Samples with incorrect information were excluded. (3)Samples with outliers were excluded. This paper finalized the sample size of 20,075 individuals was added

### Variables selection

Explained variable: the residents’ perceptions of social fairness. In this paper, we use perceptions of social fairness indicators from the 2013, 2015, and 2017 CGSS.The questionnaire of China General Social Survey asked respondents “In general, do you think the society today is fair?” The results were measured on a five-point Likert scale: Very unfair = 1, relatively unfair = 2, cannot say fair not fair = 3, relatively fair = 4, very fair = 5.

Explanatory variable: local government competition. Local government competition data was from the China Statistical Yearbook and the National Economic and Social Development Statistical Bulletin. Given that the location of the household micro-data selected for this paper can only be located at the provincial level. This paper will be based on the province where the household is located. Following Zhang et al. (2007), the ratio of foreign direct investment at the provincial level to the number of permanent residents in the area is used to measure local government competition.

Mediating variables: income disparity, basic government public goods, and corruption. Corruption data indicators were from China Prosecution Yearbook, Thiel coefficient and government supply of basic public goods data indicators were from China Statistical Yearbook. The income gap indicator uses the Thayer coefficient. The government basic public goods indicator refers to Li et al. (2018) and is measured by the *per capita* social spending in the corresponding area of the respondent. *Per capita*, social spending mainly refers to the total *per capita* investment of local governments in education, health care, social security, and employment. Corruption indicators refer to the existing literature. He et al. (2016), use the number of office crimes filed per 10,000 public officials to measure the degree of corruption in each region.

Control variables: To reduce the possible bias of model estimation caused by omitted variables, the following control variables are introduced in this paper in combination with existing literature. The control variables here were all from the CGSS form2013, 2015, and 2017.These include: (1) gender (Female = 0, Male = 1). (2) Age (Age of respondents). (3) Household registration (Rural = 0, Town = 1), (4) Ethnicity (Other = 0, Han = 1), (5) Education level (1 to 7, the higher the value, the higher the level of education), (6) Political appearance (Other = 0, Party member = 1). (7) Marital status (Other = 0, Married = 1). (8) Health status (1 to 5, the higher the value, the healthier the self-assessment), (9) Self-assessed social status (1 to 10, the higher the value, the higher the self-rated social status), (10) Household income (income is taken as logarithm), (11) Self-assessed family status (1 to 5, the higher the value, the higher the self-rated household economic status; Table 1).

**Table 1:** Descriptive statistics of main variables.

**Variables**	**Description**	**Mean**	**SD**	**Min**	**Max**
fair	social perceptions of fairness	3.11	1.06	1	5
gov	local government competition	5.24	1.09	2.82	7.02
gap	income gap	0.02	0.05	−0.059	0.15
fuwu	supply of basic public goods	0.38	0.04	0.31	0.48
corrpt	corruption	18.87	7.71	4.62	34.38
gender	gender	0.48	0.50	0	1
age	age	51.04	16.70	18	103
hukou	hukou	0.47	0.50	0	1
nation	ethnicity	0.92	0.27	0	1
edu	education	2.84	1.63	1	14
party	politics face	0.12	0.32	0	1
marry	marriage situation	0.76	0.43	0	1
health	health status	3.49	1.09	1	5
status	self-assessment of social status	4.16	1.68	1	10
income_family	household income	10.68	1.24	1.94	16.12
economy_family	self-assessment of family economic status	2.55	0.75	1	5

### Model settings

We developed an overall model to measure the impact of local government competition on residents’ perceptions of social fairness. Based on the previous analysis, the following model is developed for empirical analysis:


(1) $$fair_{it}=\alpha 0+\alpha 1gov_{it}+\beta \;\Sigma x_{it}+u_{it}$$

fairit indicates the perceptions of social fairness of the surveyed residents in different provinces *i* and year *t*, govit indicates the degree of local government competition at the provincial level, xit is other control variables, uit is a random disturbance term, and α0,α1 and β are regression coefficients. Since the explained variable perceptions of social fairness is a multinomial ordered choice variable, this paper chooses the ordered probit (Ordered Probit) model for parameter estimation.

To further investigate its specific mechanism of action and verify the mechanistic framework of the theoretical analysis part, the following test steps are designed in this paper: firstly, test the effect of local government competition on residents’ subjective perceptions of social fairness according to the equation. (2) Secondly, test the existence of mediating effect from the effect of local government competition on each mediating variable according to the equation. (3) Finally, add both local government competition variables and mediating variables in the regression equation to test the inner mechanism of the effect of local government competition on residents’ perceptions of social fairness, as shown in equation (4).


(2) $$fair_{it}=\alpha 0+\alpha 1gov_{it}+\beta \;\Sigma x_{it}+u_{it}$$

(3) $$P=\gamma 0+\gamma 1gov_{it}+\gamma \;\Sigma x_{it}+u_{it}$$

(4) $$fair_{it}=\delta 0+\delta 1gov_{it}+\rho P+\delta \;\Sigma x_{it}+u_{it}$$

In the above equation, *i* and *t* denote province and year, respectively, and P = (p1，p2，p3) denotes the three mediating variables proposed in this paper. Uit is a random disturbance term. The data was then analyzed using statistical software stata16.

## Empirical results

### Basic regression results

Equation (1) was estimated using an ordered probit model, and the marginal effects of each variable on residents’ perceptions of social fairness were examined on this basis. The regression results are shown in Tables 2, 3, respectively.

**Table 2:** Basic regression results.

**Variables**	**(1)fair**	**(2)fair**	**(3)fair**
	**Oprobit**	**Oprobit**	**Oprobit**
gov	−0.047***	−0.065***	−0.048***
	(−4.70)	(−5.89)	(−4.22)
gender		0.030	0.032
		(1.38)	(1.44)
nation		−0.109**	−0.106**
		(−2.47)	(−2.36)
edu		0.029***	0.034***
		(3.36)	(3.91)
party		0.054	0.054
		(1.50)	(1.49)
hukou		−0.131***	−0.104***
		(−5.21)	(−3.93)
status		0.110***	0.091***
		(14.95)	(10.81)
marry		−0.082***	−0.071***
		(−3.19)	(−2.68)
age		0.012***	0.011***
		(14.91)	(12.80)
health		0.056***	0.057***
		(4.68)	(4.60)
income_family			−0.073***
			(−5.64)
economy_family			0.133***
			(7.00)
N	20,075	20,075	20,075
R2	0.108	0.219	0.242

***, **, * indicate statistically significant at the 1, 5, and 10% levels, respectively. t-values in parentheses.

**Table 3:** Regression results of marginal effects of the effects of various variables on Perceptions of social fairness.

**Variable**	**Fair = 1**	**Fair = 2**	**Fair = 3**	**Fair = 4**	**Fair = 5**
gov	0.006***	0.010***	0.002***	−0.015***	−0.003***
	(4.19)	(4.23)	(4.10)	(−4.23)	(−4.15)
gender	−0.004	−0.007	−0.001	0.010	0.002
	(−1.44)	(−1.44)	(−1.43)	(1.44)	(1.43)
nation	0.014**	0.023**	0.004**	−0.033**	−0.008**
	(2.36)	(2.36)	(2.34)	(−2.37)	(−2.34)
edu	−0.005***	−0.007***	−0.001***	0.011***	0.002***
	(−3.89)	(−3.91)	(−3.82)	(3.91)	(3.86)
party	−0.007	−0.012	−0.002	0.017	0.004
	(−1.49)	(−1.49)	(−1.49)	(1.49)	(1.49)
hukou	0.014***	0.022***	0.004***	−0.033***	−0.007***
	(3.92)	(3.93)	(3.83)	(−3.94)	(−3.87)
status	−0.012***	−0.019***	−0.003***	0.028***	0.006***
	(−10.26)	(−10.95)	(−9.34)	(10.90)	(9.93)
marry	0.009***	0.015***	0.003***	−0.022***	−0.005***
	(2.67)	(2.68)	(2.65)	(−2.68)	(−2.66)
age	−0.001***	−0.002***	−0.001***	0.003***	0.001***
	(−12.17)	(−12.82)	(−10.28)	(12.99)	(11.09)
health	−0.008***	−0.012***	−0.002***	0.018***	0.004***
	(−4.57)	(−4.61)	(−4.46)	(4.61)	(4.53)
income_family	0.010***	0.016***	0.003***	−0.023***	−0.005***
	(5.60)	(5.65)	(5.33)	(−5.67)	(−5.44)
economy_family	−0.018***	−0.028***	−0.005***	0.042***	0.009***
	(−6.91)	(−7.00)	(−6.46)	(7.01)	(6.75)
N	20,075	20,075	20,075	20,075	20,075

***, **, * indicate statistically significant at the 1, 5, and 10% levels, respectively. t-values in parentheses.

In Table 2, columns (1), (2), and (3) show the regression results without and with the inclusion of control variables, respectively. The coefficients of local government competition variables are significantly negative, implying that local government competition is not conducive to the enhancement of residents’ perceptions of social fairness. The reasons may be caused by the fact that local government competition can widen the gap between the rich and the poor, interfere with the market economy and distort the allocation of financial resources as well as increase corruption. Among the control variables ethnicity, marriage, and household registration have significantly negative coefficients, indicating that ethnic minorities, rural households, and unmarried groups have a stronger perception of social fairness. The regression coefficients of the variables education, self-rated social status, age, and health are significantly positive, which indicates that the more educated, higher self-rated social status, older, and healthier groups have a stronger perception of social justice. The regression coefficients of the variables of household income are significantly negative, which implies that higher absolute household income does not necessarily lead to higher perceptions of social fairness. The coefficient of the self-assessed household economic income status variable is significantly positive, which indicates that the higher the self-assessed household economic income status, the higher the perceptions of social fairness in the households.

In Table 3, the analysis of marginal effects shows that for each unit increase in the local government competition index, the probability of social fairness of the five categories of residents from low to high changes by 0.006, 0.01, 0.002, −0.015% and-0.003%, respectively. That is, local government competition can increase the probability of residents’ “very unfair,” “relatively unfair,” “cannot say fair or not fair,” while reducing the probability of “relatively fair” and “very fair.” This result suggests that local government competition has a suppressive effect on the increase of residents’ subjective social fairness.

### Robustness test

#### Substitution of core explanatory variables

To measure local government competition from other perspectives, referring to Miao et al. (2017), the formula is calculated as follows: a province’s economic catch-up level = (highest GDP *per capita* in neighboring provinces/province’s GDP *per capita*) × (highest GDP *per capita* in national provinces/province’s GDP *per capita*), which is estimated using the probit model according to equation (1). The results are shown in column (1) of Table 4, where the coefficient of the local government competition variable is significantly negative after replacing the measure of local government competition, and the study results remain robust.

**Table 4:** Robustness tests.

**Variables**	**(1)fair**	**(2)fair**	**(3)fair**	**(4)fair**
	**Oprobit**	**Probit**	**OLS**	**Ologit**
gov1	−0.019***			
	(2.85)			
gov		−0.040***	−0.046***	−0.081***
		(−2.77)	(−4.20)	(−4.08)
gender	0.028	0.015	0.028	0.059
	(1.26)	(0.55)	(1.35)	(1.55)
nation	−0.152***	−0.072	−0.081**	−0.167**
	(−3.41)	(−1.36)	(−1.99)	(−2.15)
edu	0.031***	0.032***	0.032***	0.059***
	(3.62)	(2.78)	(3.91)	(4.01)
party	0.060	0.007	0.048	0.089
	(1.64)	(0.16)	(1.42)	(1.37)
hukou	−0.112***	−0.102***	−0.096***	−0.176***
	(−4.16)	(−3.10)	(−3.82)	(−3.89)
status	0.093***	0.090***	0.085***	0.158***
	(10.91)	(9.34)	(11.10)	(10.81)
marry	−0.064**	−0.048	−0.062**	−0.116**
	(−2.38)	(−1.47)	(−2.50)	(−2.55)
age	0.011***	0.010***	0.010***	0.019***
	(12.32)	(9.74)	(12.76)	(12.71)
health	0.050***	0.055***	0.055***	0.102***
	(4.03)	(3.83)	(4.82)	(4.78)
income_family	−0.079***	−0.058***	−0.064***	−0.130***
	(−6.06)	(−3.75)	(−5.44)	(−5.75)
economy_family	0.132***	0.143***	0.131***	0.244***
	(6.88)	(6.39)	(7.43)	(7.49)
N	20,075	20,075	20,075	20,075
R2	0.243	0.319	0.314	0.244

***, **, * indicate statistically significant at the 1, 5, and 10% levels, respectively. t-values in parentheses.

#### Assignment method for adjusting the perceptions of social fairness

The variables have been controlled for in the selection of control variables in this paper. However, the subjective social fairness evaluation given by the respondents may not be accurate and true due to factors such as free ride motivation or respect for visitors. Based on this, the evaluation of residents’ subjective perceptions of social fairness is reassigned. The specific adjustment rules are as follows: the answers to “very fair,” “relatively fair” and “not fair or unfair” are assigned to 1, and the answers to “relatively unfair” and “unfair” are assigned to 0. “This adjustment reduces the problem of data bias caused by subjective reasons such as inconsistent standards. The estimation is then performed using a binary probit model based on the equation (1). The regression results are presented in column (2) in Table 4, and it can be found that the effect of local government competition on residents’ subjective perceptions of social fairness does not change essentially after adjusting the assignment method of the perceptions of social fairness. The coefficient of the local government competition variable remains significantly negative, which means that the research finding that local government competition amplifies residents’ perceptions of social unfairness is robust.

#### Replacement regression method

Robustness tests were conducted using Ordinary Least Squares (OLS) and Ordered Logit (OL) models. The regression results are presented in column (3) and column (4) of Table 4, which show that the regression coefficient of local government competition remains significantly negative. The results are consistent with the regression results of the Oprobit model, which indicates that the finding that local government competition is not conducive to the improvement of residents’ perceptions of social fairness is robust.

### Mechanisms

#### Income effect

This paper proposes three mechanisms by which local government competition affects residents’ perceptions of social fairness. Firstly, we examine the mechanism of the role of income disparity in local government competition in reducing residents’ perceptions of social fairness. The regression coefficient of the local government competition variable in column (2) of Table 5 is significantly positive, indicating that local government competition can widen income disparity. Further, the regression results of both the explanatory variable local government competition and the mediating variable Thayer coefficient in column (3) show that the local government competition variable is significantly negative and significantly reduced compared with the coefficient of the local government competition variable in column (1). In addition, the coefficient of the Thiel coefficient is significantly positive, implying that excessive income disparity increases residents’ perceptions of social unfairness. The regression results in columns (1), (2), and (3) show that there is a mediating effect of the Thayer coefficient between local government competition and the perceptions of social fairness, thus forming the inner mechanism of “local government competition → widening the income gap → decreasing the perceptions of social fairness.” Local government competition reduces residents’ perceptions of social fairness by widening their income gap.

**Table 5:** Results of testing the mechanism of the intrinsic effect of local government competition on residents’ perceptions of social fairness.

**Variables**	**Income Disparity**			**Basic Public Services**		**Corruption**	
	**(1)**	**(2)**	**(3)**	**(4)**	**(5)**	**(6)**	**(7)**
	**fair**	**tier**	**fair**	**fuwu**	**fair**	**corrpt**	**fair**
gov	−0.048***	0.016***	−0.052***	−0.025***	−0.026*	2.617***	−0.026**
	(−4.22)	(5.23)	(−4.32)	(−5.83)	(−1.80)	(−3.45)	(−2.13)
tier			0.244**				
			(1.12)				
fuwu					−0.900**		
					(2.56)		
corrupt							−0.009***
							(5.42)
gender	0.032	0.001	0.032	−0.000	0.032	0.031	0.032
	(1.44)	(0.87)	(1.43)	(−0.10)	(1.44)	(0.23)	(1.43)
nation	−0.106**	−0.005***	−0.105**	0.024***	−0.128***	4.072***	−0.142***
	(−2.36)	(−3.69)	(−2.34)	(14.13)	(−2.81)	(15.40)	(−3.11)
edu	0.034***	0.001***	0.033***	−0.000*	0.034***	−0.153***	0.035***
	(3.91)	(3.41)	(3.86)	(−1.73)	(3.96)	(−2.78)	(4.07)
party	0.054	−0.006***	0.056	0.001	0.053	−0.043	0.055
	(1.49)	(−3.23)	(1.53)	(1.14)	(1.46)	(−0.20)	(1.51)
hukou	−0.104***	0.016***	−0.108***	−0.009***	−0.096***	−1.123***	−0.095***
	(−3.93)	(13.68)	(−4.06)	(−10.48)	(−3.62)	(−6.43)	(−3.58)
status	0.091***	−0.000	0.091***	0.000	0.091***	−0.102**	0.092***
	(10.81)	(−0.43)	(10.81)	(0.48)	(10.81)	(−2.10)	(10.92)
marry	−0.071***	−0.005***	−0.069***	0.003***	−0.073***	0.560***	−0.075***
	(−2.68)	(−3.60)	(−2.63)	(4.03)	(−2.79)	(3.48)	(−2.86)
age	0.011***	0.001***	0.011***	−0.001	0.011***	−0.018***	0.011***
	(12.80)	(4.44)	(12.72)	(−0.25)	(12.81)	(−3.27)	(12.96)
health	0.057***	−0.001**	0.057***	0.002***	0.056***	−0.113	0.058***
	(4.60)	(−2.07)	(4.63)	(4.57)	(4.49)	(−1.54)	(4.69)
income_family	−0.073***	0.004***	−0.074***	−0.005***	−0.069***	−0.828***	−0.066***
	(−5.64)	(7.74)	(−5.70)	(−13.08)	(−5.27)	(−10.33)	(−5.10)
economy_family	0.133***	−0.002**	0.133***	0.003***	0.130***	0.294***	0.130***
	(7.00)	(−2.52)	(7.03)	(5.10)	(6.86)	(2.64)	(6.87)
N	20,075	20,075	20,075	20,075	20,075	20,075	20,075
R2	0.240	0.193	0.245	0.467	0.251	0.216	0.259

***, **, * indicate statistically significant at the 1, 5, and 10% levels, respectively. t-values in parentheses.

#### Public service effects

Then, the mechanism of the role of government basic public goods supply in local government competition in reducing the perception of social fairness is examined. The coefficient of local government competition in column (4) of Table 5 is significantly negative, indicating that local government competition can reduce the supply of basic government public goods. Further, column (5) puts both the explanatory variable of local government competition and the mediating variable of government supply of essential public goods. The regression results show that the coefficient of local government competition is significantly negative and decreases significantly compared with the coefficient of local government competition in column (1), and the coefficient of government supply of basic public goods is significantly positive, which means that government supply of basic public services can improve residents’ perceptions of social fairness. The regression results in columns (1), (4), and (5) show that there is a mediating effect of government supply of basic public goods in the relationship between local government competition and perception of social fairness, thus forming an internal mechanism of “local government competition → reduction of government supply of basic public goods → reduction of perception of social fairness.” Local government competition leads the government to invest financial resources in infrastructure projects for the sake of economic growth while squeezing out the supply of basic government public goods such as public education, health care, and social security, thus increasing residents’ perceptions of social unfairness.

#### Corruption effect

Finally, the mechanism of the role of corruption in local government competition in reducing residents’ perception of social fairness is examined. The regression coefficient of the local government competition variable in column (6) of Table 5 is significantly positive at the 1% statistical level, indicating that local government competition leads to corruption. Further, both the explanatory variable local government competition, and the mediating variable corruption are placed in column (7). The regression results show that the coefficient of the local government competition variable is significantly negative and significantly reduced compared to the coefficient of the local government competition variable in column (1). In addition, the coefficient of corruption is significantly negative, implying that corruption inhibits the perception of fair social fairness. In summary, the regression results in columns (1), (6), and (7) show that there is a mediating effect of corruption between local government competition and residents’ perceptions of social fairness, thus forming an internal mechanism of “local government competition → increased corruption → decreased perceptions of social fairness.” That is, local government competition increases corruption and thus decreases residents’ perceptions of social fairness. The effect of local government competition increases corruption and thus reduces residents’ perceptions of social fairness.

## Heterogeneity analysis

There may be group heterogeneity in the effect of local government competition on the perceptions of residents’ social fairness. This paper further analyzes the heterogeneity of the effect of local government competition on the perception of social fairness across different income groups, urban and rural groups, and geographic groups. The regression results are shown in Table 6.

**Table 6:** Heterogeneity test results of the effect of local government competition on the perception of social fairness.

**Variables**	**(1)**	**(2)**	**(3)**	**(4)**	**(5)**	**(6)**	**(7)**	**(8)**
	**Low-income**	**Middle-income**	**High-income**	**Rural**	**Towns**	**East**	**Central**	**West**
gov	−0.035	−0.062***	−0.050***	−0.033*	−0.053***	−0.049***	−0.030***	−0.120***
	(−1.50)	(−3.17)	(−2.58)	(−1.86)	(−3.40)	(−2.91)	(−1.12)	(−3.60)
gender	0.072*	0.000	−0.003	0.032	0.010	0.061*	0.051	−0.059
	(1.69)	(0.00)	(−0.09)	(1.03)	(0.32)	(1.89)	(1.20)	(−1.29)
nation	−0.107	−0.070	−0.220**	−0.152***	−0.077	−0.016	−0.280***	−0.130
	(−1.49)	(−0.88)	(−2.41)	(−2.61)	(−1.05)	(−0.26)	(−2.70)	(−1.34)
edu	0.063***	0.022	0.004	0.055***	0.003	0.021*	0.035**	0.041**
	(3.50)	(1.36)	(0.27)	(3.66)	(0.30)	(1.70)	(2.06)	(2.29)
party	0.079	−0.000	0.140***	0.033	0.112**	0.061	0.176**	−0.047
	(0.73)	(−0.01)	(2.77)	(0.48)	(2.50)	(1.25)	(2.22)	(−0.59)
hukou	−0.129**	−0.089**	−0.022	——	——	−0.161***	−0.149***	−0.016
	(−2.54)	(−2.02)	(−0.43)			(−3.90)	(−3.05)	(−0.32)
status	0.079***	0.103***	0.096***	0.076***	0.115***	0.102***	0.092***	0.078***
	(5.40)	(7.18)	(6.49)	(6.73)	(9.02)	(8.41)	(5.56)	(4.57)
marry	−0.127***	−0.057	0.025	−0.111***	−0.005	−0.039	−0.086	−0.075
	(−2.76)	(−1.15)	(0.54)	(−2.83)	(−0.14)	(−1.01)	(−1.64)	(−1.37)
age	0.016***	0.010***	0.006***	0.015***	0.006***	0.010***	0.012***	0.013***
	(10.40)	(6.48)	(3.89)	(12.17)	(5.01)	(7.62)	(7.26)	(7.07)
health	0.049**	0.059***	0.049**	0.053***	0.049**	0.029	0.086***	0.056**
	(2.37)	(2.69)	(2.14)	(3.23)	(2.56)	(1.62)	(3.42)	(2.21)
income_family	−0.069***	−0.064**	−0.027	−0.063***	−0.057**	−0.057***	−0.103***	−0.058**
	(−3.64)	(−2.04)	(−0.86)	(−3.91)	(−2.51)	(−2.87)	(−4.17)	(−2.20)
economy_family	0.162***	0.138***	0.103***	0.131***	0.133***	0.138***	0.125***	0.122***
	(4.80)	(4.11)	(3.15)	(5.05)	(4.64)	(4.98)	(3.39)	(3.14)
N	6,491	6,756	6,828	12,841	7,234	8,163	5,689	6,233
R2	0.341	0.250	0.196	0.272	0.255	0.261	0.307	0.220

***, **, * indicate statistically significant at the 1, 5, and 10% levels, respectively. t-values in parentheses.

### Heterogeneity analysis of different income groups

The degree of perception of social fairness is not consistent across income class groups. To examine whether there are differences in the effects of local government competition on the Perceptions of social fairness among different income class groups, all samples are divided into three groups in this section, with the first one-third being the low-income sample, the middle being the middle-income sample, and the second one-third being the high-income sample according to the principle of three equal annual personal income, and then regressed using an ordered probit model. The results are shown in Table 6, in which the coefficients of local government competition variables in columns (1) and (2) are significantly negative, and the coefficients of local government competition variables in column (3) are not significant, which can be seen that local government competition has some differences on the social fairness Perceptions of individual residents in different income groups. In general, local government competition has a significant negative effect on the social fairness of middle-income and high-income groups, but not on the social fairness of low-income groups. The possible reason is that local government competition helps to increase the income of high-income social groups to the detriment of low-income social groups, and the effect of local government on increasing the income of high-income groups is more prominent (Jia and Liang, 2020). In addition, social benefits provided by the government, such as basic public services, are often characterized by status and privilege in China, with higher benefits for higher-income groups, and the bottom-class workers or migrant workers, who make up the majority of China’s population, do not enjoy the benefits of public systems such as social security. Precisely because low-income groups have lower levels of access to income-increasing effects and public services, they are not as sensitive as high-income groups to income-increasing effects and basic public government services, and thus local governments have a more significant impact on the perceptions of social fairness of the middle-income and above groups.

### Urban–rural heterogeneity analysis

The urban–rural dichotomy can have different effects on local government behavior and the social attitudes of residents in the jurisdiction. To explore whether there is a difference in the perception of social fairness of local government competition for different household registration groups, this section divides the entire sample into urban and rural groups according to household registration, on which equation (1) is estimated using an ordered probit model. The regression results are shown in columns (4) and (5) of Table 6. The regression results are shown in columns (4) and (5) of Table 6, and the coefficients of the local government competition variables are significantly negatively correlated for individuals with different household registration. However, in general, local government competition has a greater impact on the perception of social fairness for urban residents than for rural residents. This phenomenon may be due to the fact that while local government competition increases the level of urban development, economic development has an impact on the population’s expectations of social mobility. It has been shown that economic development has an impact on residents’ perceptions of social fairness through the inherent transmission mechanism of social mobility expectations. In the process of modern social transformation and urban upgrading, rapid economic growth has expanded regional mobility, and the rural working population has successively flocked to the cities, receiving more job opportunities and opportunities for horizontal mobility with regional transfers. In contrast, urban residents have fewer opportunities for horizontal mobility, and the social mobility expectations of rural household residents and farmers are higher than those of urban residents, thus triggering negative and complaining urban residents about their perceptions of the community (Samuel and Huntington, 2015).

### Regional heterogeneity analysis

There are large disparities in the degree of local government competition across regions. To examine whether there are differences in the effects of local government competition on residents’ perceptions of social fairness in different regions, this section further divides the entire sample into eastern, central, and western regions, on which equation (1) is estimated using an ordered probit model. The regression results are shown in columns (6), (7), and (8) of Table 6. The coefficients of the local government competition variables in different regions are significantly negative, indicating that local government competition significantly impairs the perceptions of social equality of residents in each region, but among them, it has the greatest impact on the perceptions of social equality in the eastern region and the least impact in the central and central-western regions. This may be because local government competition exacerbates interregional inequality in public services. In a system of political centralization and economic decentralization, the central government’s transfer policy becomes a tool to manage the behavior of local governments smoothing the inter-regional ability to pay, with the greatest inequality in public services in the eastern region, followed by the central region, while public services in the western region are generally less equal and relatively balanced than those in the east (Zhao and Fu, 2017).

## Discussion

Based on micro-survey data from the years 2013, 2015, and 2017 China General Social Survey (CGSS) and macro-government level data. This paper empirically analyzes the effect and mechanism of local government competition on residents’ subjective perceptions of social fairness using an ordered probit model. The main findings of this paper are: (1) local government competition has a significant inhibitory effect on residents’ subjective social fairness perceptions, and this finding still holds after a series of tests. (2) In terms of mechanism of action, local government competition expands residents’ subjective social unfairness through widening income disparity, affecting the supply of basic government public goods, and increasing corruption. (3) Local government competition has a significant negative effect on the social fairness perceptions of the middle-income as well as the high-income groups but does not affect low-income groups. (4) For rural residents, the effect of local government competition on urban residents’ subjective perceptions of social justice is greater. (5) There are differences in the effects of local government competition on residents’ subjective perceptions of social justice in different regions, among which residents in eastern and central regions are more affected than those in western regions.

## Conclusion

By analyzing the above findings, this paper draws the following insights:

Firstly, this study finds that local government competition in a “promotion tournament” centered on economic development reduces residents’ sense of social equity. Therefore, it is necessary to change the one-dimensional competition model with economic development as the core indicator in the “promotion tournament.” Improve the competition mechanism and assessment mode of local governments, and participate in the competition with different objectives, so as to build a multiple competition mode of social fairness and economic development. Improving the government’s governance capacity and competition mechanism promotes the consistency and balance between competing goals from a long-term perspective when local governments actively pay attention to the social and livelihood demands of residents in their jurisdictions.

Secondly, this study finds that local government competition reduces residents’ sense of social equity through the path of crowding out the government’s supply of essential public goods. Therefore, local governments should optimize the structure of public expenditure. The government’s supply of basic public services has a non-negligible impact on people’s perceptions of social fairness. The government should increase its financial investment in the supply of basic public goods and further improve the equalization of basic public services such as education, health care, and social security among different income groups through system reform, especially the investment in social welfare for the bottom group is especially important, which will help improve different social groups’ perceptions of social access to public welfare and perceptions of social fairness.

Thirdly, this paper finds that local government competition reduces residents’ sense of social justice by widening the income between residents. Therefore, the government should further narrow the gap between the rich and the poor, and build a harmonious and fair society of common prosperity. Excessive income disparity is a challenge that has to be faced in the process of achieving common wealth, and income disparity is still a major factor affecting social justice. The government should strengthen income distribution and regulation policies to optimize resource allocation and coordinate urban–rural and regional economic development, and reduce the urban–rural income gap and regional income gap so that the public can share the economic fruits of reform and opening up.

Fourthly, this study finds that local governments amplify residents’ sense of social inequity through corruption. Therefore, the government should insist on continuous anti-corruption sustained adherence to anti-corruption should never be a phase, but rather a policy tool that should be sustained. Corruption curbs private investment and reduces the efficiency of public investment, and the resulting social inequality and low income of the population can cause public discontent. The government should insist on anti-corruption and strengthen anti-corruption institution building and propaganda, but also can improve residents’ perception of social fairness by directing the public’s attention from an anti-corruption performance such as specific corruption cases, amounts, and numbers, to the effect of anti-corruption on narrowing income disparity and promoting social fairness.

Finally, the negative effects of economic competition among local governments should not be blamed and denied, but the most important thing is to build a reasonable and orderly competition model for local governments and to motivate and mobilize local governments to participate in the competition to achieve the goal of coordinated economic and social development in the system design.

## Data availability statement

The original contributions presented in the study are included in the article/Supplementary material; further inquiries can be directed to the corresponding author.

## Author contributions

JY conceived and designed this study, analyzed the data, and wrote the paper. JL completed conception and the funding acquisition. All authors contributed to the article and approved the submitted version.

## Funding

This work was supported by the Youth Program of the National Social Science Foundation of China (No. 17CGL013).

## Conflict of interest

The authors declare that the research was conducted in the absence of any commercial or financial relationships that could be construed as a potential conflict of interest.

## Publisher’s note

All claims expressed in this article are solely those of the authors and do not necessarily represent those of their affiliated organizations, or those of the publisher, the editors and the reviewers. Any product that may be evaluated in this article, or claim that may be made by its manufacturer, is not guaranteed or endorsed by the publisher.
